# Gamma-glutamyl transferase and risk of all-cause and disease-specific mortality: a nationwide cohort study

**DOI:** 10.1038/s41598-022-25970-0

**Published:** 2023-01-31

**Authors:** Eun Ju Cho, Su-Min Jeong, Goh Eun Chung, Jeong-Ju Yoo, Yuri Cho, Kyu-na Lee, Dong Wook Shin, Yoon Jun Kim, Jung-Hwan Yoon, Kyungdo Han, Su Jong Yu

**Affiliations:** 1 Department of Internal Medicine and Liver Research Institute, Seoul National University College of Medicine, Seoul, Republic of Korea; 2 Department of Medicine, Seoul National University College of Medicine, 101 Daehak-No, Jongno-Gu, Seoul, 03080 Republic of Korea; 3 Department of Family Medicine, Seoul National University Health Service Center, Seoul, Republic of Korea; 4 Department of Family Medicine, Seoul National University Hospital, Seoul, Republic of Korea; 5 Department of Internal Medicine and Healthcare Research Institute, Seoul National University Hospital Healthcare System Gangnam Center, Seoul, Republic of Korea; 6 Department of Internal Medicine, Division of Gastroenterology and Hepatology, Soonchunhyang University Bucheon Hospital, Gyeonggi-do, Republic of Korea; 7 Center for Liver and Pancreatobiliary Cancer, National Cancer Center, Goyang, Republic of Korea; 8 Department of Biomedicine and Health Science, The Catholic University of Korea, Seoul, Republic of Korea; 9 Department of Family Medicine/ Supportive Care Center, Samsung Medical Center, Sungkyunkwan University School of Medicine, Seoul, Republic of Korea; 10 Department of Clinical Research Design and Evaluation/Department of Digital Health, Samsung Advanced Institute for Health Science, Seoul, Republic of Korea; 11 Department of Statistics and Actuarial Science, Soongsil University, 369 Sangdo-Ro, Dongjak-Gu, Seoul, 06978 Republic of Korea

**Keywords:** Diseases, Medical research

## Abstract

Population-based data regarding the prognostic implication of gamma-glutamyl transferase (GGT) have been inconsistent. We examined the association of GGT with all-cause and disease-specific mortality. Using the Korean nationwide database, we included 9,687,066 subjects without viral hepatitis or cirrhosis who underwent a health examination in 2009. Subjects were classified into three groups by sex-specific tertile of serum GGT levels. The underlying causes of death were classified by 10th Revision of the International Classification of Diseases codes. During the median follow-up period of 8.3 years, 460,699 deaths were identified. All-cause mortality increased as serum GGT levels became higher (hazard ratio [HR], 95% confidence interval [CI] 1.05, 1.04–1.05 in the middle tertile, and 1.33, 1.32–1.34 in the high tertile) compared to the low tertile of serum GGT levels. Similar trends were observed for cardiovascular disease (CVD) (HR, 95% CI 1.07, 1.05–1.09 in the middle tertile, 1.29, 1.26–1.31 in the high tertile), cancer (HR, 95% CI 1.08, 1.07–1.10 in the middle tertile, 1.38, 1.36–1.39 in the high tertile), respiratory disease (HR, 95% CI 1.10, 1.08–1.13 in the middle tertile, 1.39, 1.35–1.43 in the high tertile), and liver disease mortality (HR, 95% CI 1.74, 1.66–1.83 in the middle tertile, 6.73, 6.46–7.01 in the high tertile). Regardless of smoking, alcohol consumption and history of previous CVD and cancer, a higher serum GGT levels were associated with a higher risk of mortality. Serum GGT levels may be useful for risk assessment of all-cause and disease-specific mortality in general population.

## Introduction

Gamma-glutamyl transferase (GGT), a key enzyme in the metabolism of glutathione, performs important roles in antioxidant mechanism^1^. Given its role as a major antioxidant, GGT has been known as a surrogate marker of oxidative stress, and various studies have reported the involvement of GGT in the pathogenesis of various diseases, such as cardiovascular disease (CVD), cancer, lung inflammation, and neurologic diseases^2^.

Serum GGT level has also been known as a predictor of mortality in the general population. GGT level even within the reference range was associated with an increased all-cause and cardiovascular mortality after adjusting for confounding factors^3–5^. However, except cardiovascular mortality, few studies showed data on the disease-specific mortality which hampered detailed analysis of the association of GGT level and other disease-specific death in the general population^6^. In addition, the size of the study cohorts was frequently limited, and they were usually from Western countries^4,6,7^. In addition, GGT is undoubtedly regarded as an indicator for liver function and alcohol consumption. However, many of previous studies did not consider underlying liver diseases or alcohol drinking habits, which may distort the results^8,9^.

Until now, the utility of GGT in the prognostication of general population regarding disease-specific mortality had not been fully established in Asia. Thus, we aimed to provide a comprehensive analysis of both all-cause and disease-specific mortality according to GGT levels and their extra-hepatic implications, using a nationwide population-based cohort in South Korea.

## Methods

### Data source and study setting

We used an anonymized data from the National Health Insurance Service (NHIS) of Korea. As Korea has national health insurance system provided by single government insurer, NHIS covers almost entire population (more than 97%), while the remaining 3% in the low-income bracket are covered by the Medical Aid program. The NHIS database includes claims of medical utilization for reimbursement including medical treatments and procedures, and disease diagnoses according to the International Classification of Disease, 10th revision (ICD-10)^10^. Furthermore, NHIS offers biennial National Health Screening Program (NHSP) for all insured members, which consisted of a self-reported questionnaire on lifestyle behavior, anthropometric measurements, and laboratory testing^11^.

### Study population

**Figure 1:**
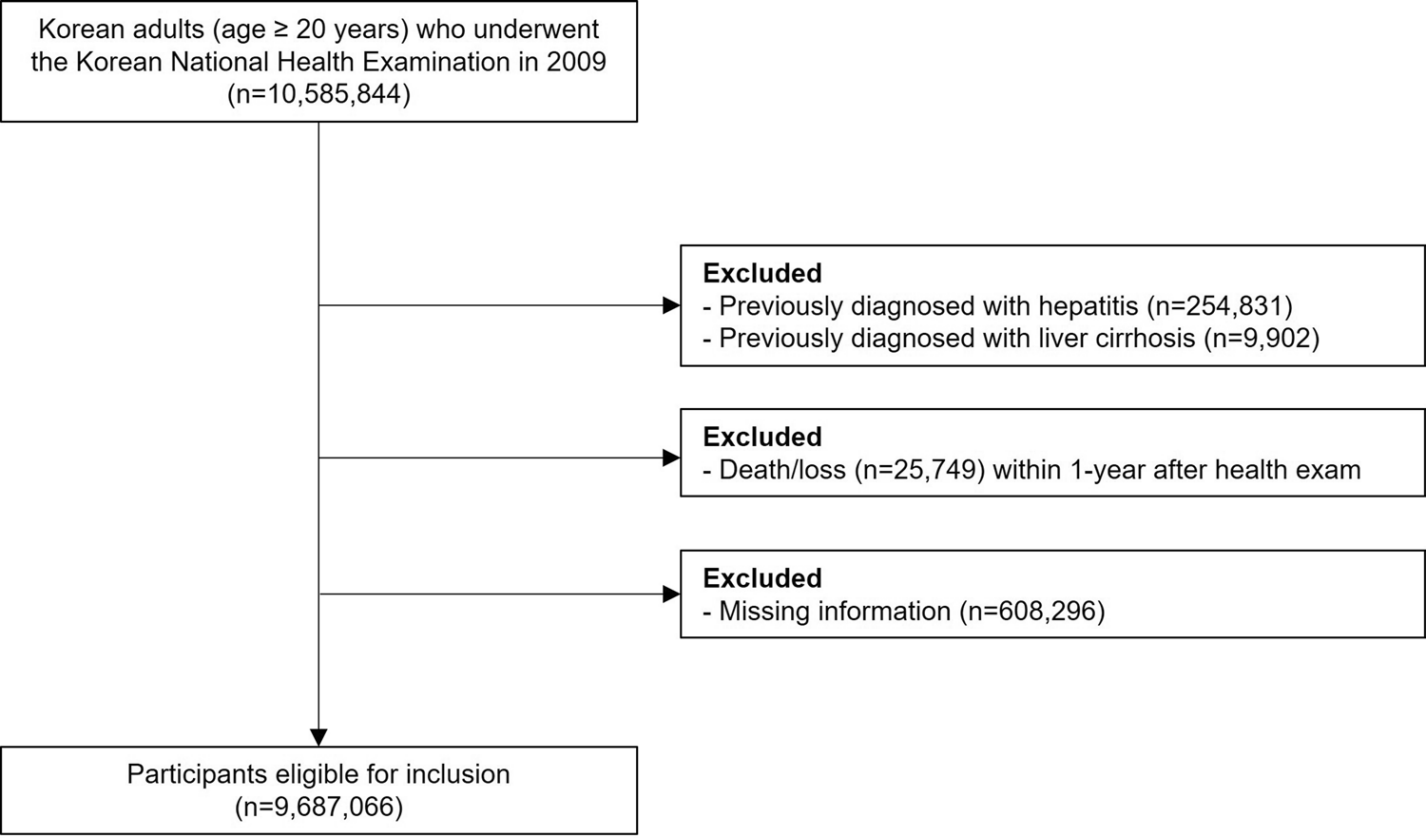
Flow chart of the study population.

We initially included 10,585,844 Korean adults aged 20 years or older who had participated in NHSP in 2009 from the NHIS database of the entire Korean population. The participants with previously diagnosed with viral hepatitis (ICD-10: B15-18) (n = 254,831) or liver cirrhosis (ICD-10: K74) (n = 9,902) before 2009 were excluded. We also excluded those who died within the first year of the follow-up period (n = 25,749): 1-year lag time was applied to minimize the reverse causality. After excluding those with missing information (n = 608,296) (Supplementary Table S1), we finally included 9,687,066 (Fig. 1). The participants were followed up from 1 year after the day of NHSP until December 31. 2019, or date of death, whichever came first.

The Institutional Review Board of Soongsil University approved this study (SSU-202007-HR-236-01), and we performed the study in accordance with relevant guidelines and regulations. The requirement for written informed consent was waived by the Institutional Review Board of Soongsil University due to the use of anonymous and de-identified information.

### Measurement and definitions

From laboratory results, serum levels of GGT, alanine transaminase (ALT), aspartate aminotransferase (AST), glucose, and lipid profile were obtained on the day of health check-up. Level of serum GGT was categorized by sex-specific tertiles as follows: low tertile (< 25 IU/L), middle tertile (25–43 IU/L), and high tertile (≥ 44 IU/L) for men and low tertile (< 14 IU/L), middle tertile (14–20 IU/L), and high tertile (≥ 21 IU/L) for women.

Through health exam (NHSP), height, weight, waist circumference, and blood pressure were measured, and body mass index (BMI) was calculated as weight (kg) divided by height (m^2^). Smoking status, alcohol drinking habits, and physical activity were assessed by self-reported questionnaires. Smoking status was classified into non-, ex-, and current smoker. The amount of alcohol consumption was categorized into none, mild to moderate, and heavy (≥ 30 g/day for men and 20 g/day for women). Regular physical activity was defined as when participants meet one of the following two criteria: (1) Engaging in physical activity with vigorous intensity at least 20 min/day for 3 or more days/week, or (2) Engaging in physical activity with moderate intensity during at least 30 min/day for 5 or more days/week.

To define comorbidities such as diabetes mellitus, hypertension, and dyslipidemia, we used a combination of information from NHSP and ICD-10 codes for disease with prescription history of relevant medication: diabetes mellitus was defined with a fasting glucose level of at least 126 mg/dL and prescription history of hypoglycemic agents and insulin with ICD-10 codes (E11–14). Hypertension was defined as elevated blood pressure (systolic blood pressure ≥ 140 mmHg or diastolic pressure ≥ 90 mmHg) and prescription history of antihypertensive medications with ICD-10 codes (I10–13 and I15). Dyslipidemia was defined as a total cholesterol level greater than 240 mg/dL and prescription history of lipid-lowering medications with ICD-10 code (E78). The Charlson comorbidity index (CCI) was assessed by ICD-10 codes and was treated as a continuous variable^12^.

### Outcome: Causes of death

Statistics Korea provides death data and cause of death based on death certificates, and this information was linked to NHIS database. Statistics Korea gathered information on death and cause of death based on death certificates or postmortem examination certificates issued by physicians who directly examined the person^13^. Physicians judged cause of death considering underlying disease based on previous medical history. Causes of death were selected considering the leading causes of death in Korea. Identification of cause of death was based on the Korean Standard Classification of Diseases and Causes of Death, which corresponds to ICD-10 codes. Death caused by CVD (I00–I99), malignant neoplasm (C00–C97), respiratory disease (J00–J99), and liver disease (K70–K76) was identified. Mortality from liver cancer was included in cancer-specific mortality, not in liver disease.

### Statistical analysis

Differences among tertiles of GGT were confirmed by one-way analysis of variance for continuous variables and Chi-square tests for categorical variables. Continuous variables were presented as mean ± standard deviation (SD) values for normally distributed variables and geometric mean with 95% confidence interval (CI) values for skewed variables (e.g., triglyceride, ALT, AST, and GGT). Categorical variables were presented as numbers with proportions.

According to sex-specific tertiles, number of deaths and incidence rates for all cause death and disease-specific death (per 1000 person-years) were assessed. Cox proportional hazards analyses were performed to examine the association between GGT level and mortality risk, adjusted for age and sex (model 1), lifestyle behaviors (smoking status, alcohol consumption, and physical activity), and income level (model 2) and additionally BMI, ALT, comorbidities (diabetes mellitus, hypertension, and dyslipidemia), and CCI score (model 3). In sensitivity analyses, we performed analyses with (1) additional covariates fatty liver index (FLI) and AST/ALT ratio, (2) 2-year and 4-year lag time, and (3) quintiles of GGT. Furthermore, stratified analysis by sex, age (< 40, 40–64, and ≥ 65), smoking status, alcohol consumption, and history of CVD and cancer was conducted for all-cause and disease-specific mortality. SAS version 9.4 (SAS Institute, Cary, NC, USA) was used and a two-tailed *p*-value less than 0.05 was regarded as statistically significant.

## Results

### Baseline characteristics

Table 1 depicts baseline characteristics of the study population by tertile of serum GGT level according to sex. The low GGT tertile group included a larger number of young participants (< 40 years old) than the middle and high tertile groups in both sexes (42.9% of male and 35.5% of female). The participants in the low tertile tended to be non-smokers and non-alcohol drinkers, especially in males, whereas most females across the tertiles were non-smokers and non-drinkers. In addition, current smokers in the high tertile group were more likely to be heavy smokers (≥ 20 pack-years) than other tertiles. Those in the high tertile group were more likely to have comorbidities including diabetes mellitus, hypertension, and dyslipidemia than other groups. The mean values of BMI, waist circumference, blood pressure, fasting glucose, total cholesterol, and liver enzymes (AST and ALT) increased with serum GGT tertile. FLI increased as GGT tertile became higher. The prevalence of an FLI of 60 or more in the low tertile was 1.6%, 11.5% in the middle tertile, and 47.7% in the high tertile. The AST/ALT ratio decreased as GGT tertile increased.

**Table 1:** Baseline characteristics according to tertiles of gamma-glutamyl transferase (GGT) by sex.

GGT	Male			Female		
	Tertile 1 (low)	Tertile 2 (middle)	Tertile 3 (high)	Tertile 1 (low)	Tertile 2 (middle)	Tertile 3 (high)
	(n = 1,761,605)	(n = 1,736,845)	(n = 1,778,947)	(n = 1,355,231)	(n = 1,593,397)	(n = 1,461,041)
Age, years						
< 40	755,515 (42.9)	618,453 (35.6)	566,864 (31.9)	481,579 (35.5)	400,456 (25.1)	201,197 (13.8)
40–64	769,684 (43.7)	920,496 (53.0)	1,058,180 (59.5)	728,795 (53.8)	944,934 (59.3)	968,649 (66.3)
≥ 65	236,406 (13.4)	197,896 (11.4)	153,903 (8.6)	144,857 (10.7)	248,007 (15.6)	291,195 (19.9)
Smoking						
Non	658,945 (37.4)	523,782 (30.2)	418,063 (23.5)	1,297,896 (95.8)	1,515,067 (95.1)	1,361,278 (93.2)
Former	427,114 (24.3)	445,724 (25.7)	427,053 (24.0)	26,782 (2.0)	28,403 (1.8)	27,701 (1.9)
Current	675,546 (38.4)	767,339 (44.2)	933,831 (52.5)	30,553 (2.3)	49,927 (3.1)	72,062 (4.9)
< 20PYs	486,486 (27.6)	503,089 (29.0)	558,155 (31.4)	28,770 (2.1)	45,964 (2.9)	63,627 (4.4)
≥ 20PYs	189,060 (10.7)	264,250 (15.2)	375,676 (21.1)	1,783 (0.1)	3,963 (0.3)	8,435 (0.6)
Alcohol drinking						
None	784,698 (44.5)	571,420 (32.9)	341,526 (19.2)	1,025,604 (75.7)	1,187,131 (74.5)	1,081,046 (73.9)
Mild to moderate	880,054 (50.0)	972,102 (55.9)	1,004,754 (56.5)	312,093 (23.0)	373,458 (23.4)	325,170 (22.3)
Heavy	96,853 (5.5)	193,323 (11.1)	432,667 (24.3)	17,534 (1.3)	32,808 (2.1)	54,825 (3.8)
Regular physical activity	370,185 (21.0)	349,293 (20.1)	328,578 (18.5)	203,333 (15.0)	248,957 (15.6)	234,391 (16.0)
Low income level^a^	211,157 (12.0)	202,271 (11.7)	214,301 (12.1)	263,979 (19.5)	316,357 (19.9)	291,873 (20.0)
Comorbidity						
Diabetes mellitus	111,184 (6.3)	157,507 (9.1)	247,230 (13.9)	42,154 (3.1)	91,388 (5.7)	190,416 (13.0)
Hypertension	329,036 (18.7)	457,742 (26.4)	631,552 (35.5)	188,864 (13.9)	362,570 (22.8)	529,691 (36.3)
Dyslipidemia	156,613 (8.9)	282,441 (16.3)	436,499 (24.5)	136,692 (10.1)	286,827 (18.0)	452,656 (31.0)
CCI score	0.5 ± 1.1	0.6 ± 1.1	0.6 ± 1.1	0.6 ± 1.0	0.7 ± 1.2	0.9 ± 1.4
Body mass index, kg/m^2^	22.9 ± 2.7	24.2 ± 2.9	25.2 ± 3.8	22.1 ± 2.8	23.0 ± 3.2	24.4 ± 4.1
WC, cm	80.3 ± 7.6	83.8 ± 7.9	86.4 ± 8.1	73.1 ± 8.2	75.7 ± 8.9	79.7 ± 9.5
Systolic BP, mmHg	121.5 ± 13.4	124.5 ± 13.7	128.1 ± 14.5	115.8 ± 14.4	119.4 ± 15.4	123.9 ± 16.1
Diastolic BP, mmHg	75.7 ± 9.14	77.9 ± 9.4	80.5 ± 9.9	72.0 ± 9.5	74.0 ± 9.9	76.6 ± 10.3
Fasting glucose, mg/dL	94.7 ± 20.5	98.3 ± 24.6	104.2 ± 30.2	90.9 ± 14.7	93.7 ± 18.5	100.5 ± 26.9
Total cholesterol, mg/dL	183.5 ± 36.8	195.9 ± 39.4	204.7 ± 43.3	186.2 ± 37.9	195.9 ± 41.1	206.0 ± 44.5
HDL-C, mg/dL	53.9 ± 31.5	52.9 ± 31.0	53.8 ± 32.0	60.8 ± 33.6	60.4 ± 34.6	58.7 ± 33.7
LDL-C, mg/dL	121.3 ± 282.4	119.9 ± 150.7	115.7 ± 123.7	129.3 ± 377.9	121.0 ± 169.5	123.8 ± 105.0
TG, mg/dL^b^	99.1 (99.1–99.2)	128.1 (127.9–128.2)	169.8 (169.6–169.9)	79.2 (79.1–79.3)	92.9 (92.8–92.9)	118.8 (118.7–118.9)
Remnant cholesterol, mg/dL	22.8 ± 15.2	29.7 ± 18.8	40.1 ± 26.7	18.3 ± 10.9	21.7 ± 13.4	28.0 ± 18.1
AST, IU/L^b^	21.4 (21.4–21.4)	23.9 (23.9–23.9)	30.7 (30.7–30.8)	19.1 (19.1–19.1)	20.6 (20.6–20.6)	24.7 (24.7–24.7)
ALT, IU/L^b^	18.4 (18.4–18.4)	24.2 (24.2–24.2)	35.4 (35.3–35.4)	13.7 (13.7–13.7)	16.1 (16.1–16.1)	23.1 (23.1–23.1)
GGT, IU/L^b^	17.7 (17.6–17.7)	32.1 (32.1–32.2)	79.3 (79.2–79.3)	10.6 (10.6–10.6)	16.5 (16.4–16.5)	32.7 (32.7–32.8)
Fatty liver index	17.3 ± 13.9	33.7 ± 19.5	57.1 ± 22.4	7.7 ± 8.7	14.2 ± 13.7	30.5 ± 22.0
< 30	1,481,823 (84.1)	850,913 (49.0)	251,063 (14.1)	1,312,046 (96.8)	1,401,933 (88.0)	836,701 (57.3)
30–60	251,941 (14.3)	686,991 (39.6)	678,859 (38.2)	359,567 (2.9)	169,039 (10.6)	444,161 (30.4)
≥ 60	27,841 (1.6)	198,941 (11.5)	849,025 (47.7)	3,618 (0.3)	22,425 (1.4)	180,179 (12.3)
AST/ALT ratio	1.2 (1.0–1.4)	1.0 (0.8–1.2)	0.9 (0.7–1.1)	1.4 (1.2–1.7)	1.3 (1.1–1.5)	1.07 (0.9–1.3)

^a^Lower 20% of income.

^b^Geometric mean with 95% confidence interval.

Values are presented as mean ± standard deviation or median (range) for continuous variables and number (%) for categorical variables.

CCI, Charlson comorbidity index; WC, waist circumference BP, blood pressure; HDL-C, high density lipoprotein-cholesterol; LDL-C, low density lipoprotein -cholesterol; TG, triglyceride; AST, aspartate transaminase; ALT, alanine transferase; GGT, gamma-glutamyl transferase.

*P-*values for all variables within each sex were < 0.001, respectively.

**Table 2:** Hazard ratios with 95% confidence interval for all-cause and disease-specific mortality by tertiles of gamma-glutamyl transferase.

GGT	Model 1	Model 2	Model 3
	HR (95% CI)		
**Total**			
All-cause mortality			
Tertile 1	1(Ref.)	1(Ref.)	1(Ref.)
Tertile 2	1.04 (1.03–1.05)	1.02 (1.01–1.02)	1.05 (1.04–1.05)
Tertile 3	1.37 (1.36–1.38)	1.30 (1.29–1.31)	1.33 (1.32–1.34)
CVD-specific mortality			
Tertile 1	1(Ref.)	1(Ref.)	1(Ref.)
Tertile 2	1.11 (1.09–1.13)	1.09 (1.07–1.11)	1.07 (1.05–1.09)
Tertile 3	1.41 (1.39–1.43)	1.37 (1.35–1.39)	1.29 (1.26–1.31)
Cancer-specific mortality			
Tertile 1	1(Ref.)	1(Ref.)	1(Ref.)
Tertile 2	1.09 (1.07–1.10)	1.06 (1.04–1.07)	1.08 (1.07–1.10)
Tertile 3	1.42 (1.40–1.44)	1.34 (1.32–1.35)	1.38 (1.36–1.39)
Respiratory disease-specific mortality			
Tertile 1	1(Ref.)	1(Ref.)	1(Ref.)
Tertile 2	0.99 (0.98–1.02)	0.99 (0.97–1.01)	1.10 (1.08–1.13)
Tertile 3	1.18 (1.15 -1.21)	1.16 (1.13–1.19)	1.39 (1.35–1.43)
Liver disease-related mortality			
Tertile 1	1(Ref.)	1(Ref.)	1(Ref.)
Tertile 2	1.70 (1.63–1.78)	1.64 (1.57–1.72)	1.74 (1.66–1.83)
Tertile 3	6.88 (6.61–7.15)	6.21 (5.96–6.46)	6.73 (6.46–7.01)
**Men**			
All-cause mortality			
Tertile 1	1(Ref.)	1(Ref.)	1(Ref.)
Tertile 2	1.04 (1.03–1.05)	1.02 (1.01–1.03)	1.07 (1.06–1.08)
Tertile 3	1.47 (1.46–1.48)	1.39 (1.37–1.40)	1.44 (1.43–1.46)
CVD-specific mortality			
Tertile 1	1(Ref.)	1(Ref.)	1(Ref.)
Tertile 2	1.13 (1.10–1.15)	1.12 (1.09–1.14)	1.09 (1.06–1.11)
Tertile 3	1.43 (1.40–1.47)	1.41 (1.37–1.44)	1.30 (1.28–1.33)
Cancer-specific mortality			
Tertile 1	1(Ref.)	1(Ref.)	1(Ref.)
Tertile 2	1.12 (1.10–1.14)	1.09 (1.07–1.11)	1.12 (1.11–1.14)
Tertile 3	1.60 (1.58–1.62)	1.49 (1.47–1.51)	1.54 (1.52–1.57)
Respiratory disease-specific mortality			
Tertile 1	1(Ref.)	1(Ref.)	1(Ref.)
Tertile 2	0.99 (0.97–1.02)	0.99 (0.96–1.02)	1.14 (1.11–1.17)
Tertile 3	1.20 (1.16–1.23)	1.18 (1.15–1.22)	1.44 (1.40–1.49)
Liver disease-related mortality			
Tertile 1	1(Ref.)	1(Ref.)	1(Ref.)
Tertile 2	1.79 (1.65–1.83)	1.68 (1.59–1.77)	1.82 (1.73–1.92)
Tertile 3	7.61 (7.28–7.96)	6.85 (6.55–7.17)	7.61 (7.27–7.97)
**Women**			
All-cause mortality			
Tertile 1	1(Ref.)	1(Ref.)	1(Ref.)
Tertile 2	0.97 (0.96–0.98)	0.97 (0.95–0.98)	0.98 (0.96–0.99)
Tertile 3	1.15 (1.13–1.16)	1.13 (1.12–1.15)	1.13 (1.12–1.15)
CVD-specific mortality			
Tertile 1	1(Ref.)	1(Ref.)	1(Ref.)
Tertile 2	1.00 (0.98–1.03)	0.99 (0.97–1.03)	0.99 (0.96–1.02)
Tertile 3	1.26 (1.23–1.29)	1.24 (1.21–1.28)	1.21 (1.18–1.25)
Cancer-specific mortality			
Tertile 1	1(Ref.)	1(Ref.)	1(Ref.)
Tertile 2	1.03 (1.00–1.05)	1.02 (1.00–1.05)	1.02 (0.99–1.04)
Tertile 3	1.19 (1.16–1.22)	1.18 (1.15–1.21)	1.14 (1.12–1.17)
Respiratory disease-specific mortality			
Tertile 1	1(Ref.)	1(Ref.)	1(Ref.)
Tertile 2	0.97 (0.93–1.01)	0.97 (0.92–1.01)	1.02 (0.97–1.06)
Tertile 3	1.12 (1.07–1.16)	1.10 (1.06–1.15)	1.24 (1.18–1.30)
Liver disease-related mortality			
Tertile 1	1(Ref.)	1(Ref.)	1(Ref.)
Tertile 2	1.37 (1.24–1.51)	1.38 (1.23–1.50)	1.38 (1.25–1.53)
Tertile 3	4.05 (3.71–4.43)	3.95 (3.62–4.32)	3.98 (3.64–4.36)

PY, person year; CVD, cardiovascular diseases, HR, hazard ratio; CI, confidence interval.

Model 1 was adjusted for age and sex; Model 2 was adjusted for smoking status, alcohol consumption, physical activity, and low income in addition to covariates in model 1; Model 3 was adjusted for body mass index, alanine aminotransferase, diabetes mellitus, hypertension, dyslipidemia, and Charlson comorbidity index score in addition to covariates in model 2.

### Association between serum GGT level and all-cause mortality

The median follow-up period was 8.3 years (interquartile range, 8.1–8.6 years). During the follow-up period, 460,699 deaths (295,567 in men and 165,132 in women) were identified among the total population, a rate of 5.8 deaths per 1,000 person-years. Among specific causes of death, cancers (mainly lung cancer) were the leading cause of death (2.0 deaths per 1000 person-years), followed by CVD (1.1 death per 1000 person-years).

Risk of CVD-, cancer-, respiratory disease-, and liver disease-specific mortality increased with serum GGT tertile (Table 2). All-cause mortality increased as serum GGT tertile increased (hazard ratio [HR], 95% CI 1.05, 1.04–1.05 in middle tertile, and 1.33, 1.32–1.34 in high tertile). Of note, a higher level of serum GGT was strongly associated with a higher risk of liver disease-related mortality (HR = 1.74, 95% CI 1.66–1.83 in middle tertile; 6.73, 6.46–7.01 in high tertile). Our results are in agreement with the association observed in the restricted cubic spline curve (Supplementary Figure S1).

### Stratified analysis: all-cause mortality by covariates

**Figure 2:**
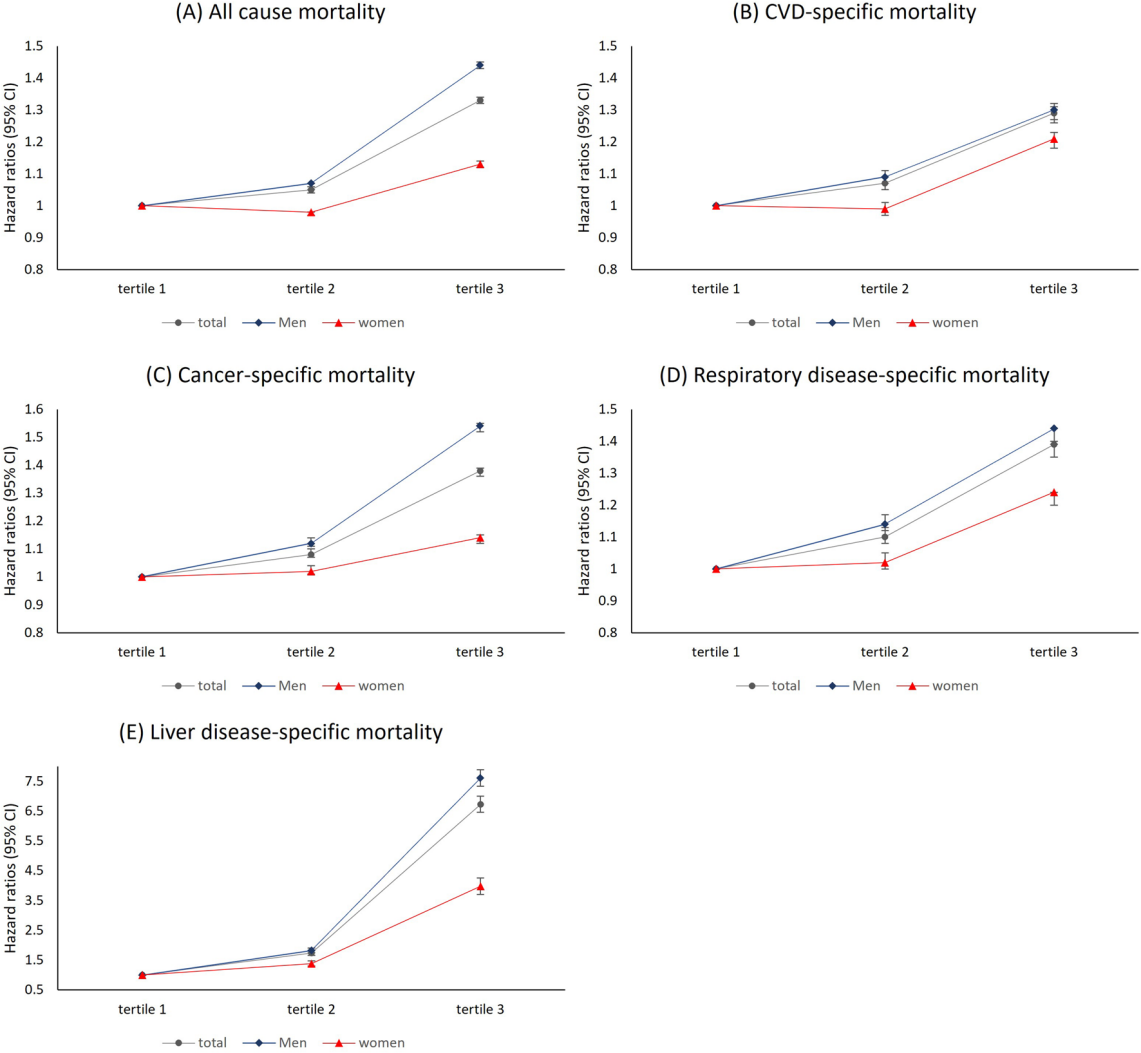
Hazard ratios (95% confidence intervals) for (**A**) all-cause, (**B**) cardiovascular disease-, (**C**) cancer-, (**D**) respiratory disease-, and (**D**) liver disease-specific mortality according to tertile of serum gamma-glutamyl transferase level

In analysis stratified by sex, stronger association between serum GGT level and mortality was found in men than women (Table 2 and Fig. 2). Among men, higher serum GGT groups had a higher all-cause mortality (HR = 1.07, 95% CI 1.06–1.08 in middle tertile; 1.44, 1.43–1.46 in high tertile) compared to the low tertile. However, women had a slightly lower all-cause mortality (HR = 0.98, 95% CI 0.96–0.99) in the middle tertile of serum GGT level and a higher all-cause mortality (HR = 1.13, 95% CI 1.12–1.15) in the high tertile compared to the low tertile.

In stratified analyses by age, a higher serum GGT level was prominently associated with a higher risk of all-cause mortality in the younger aged group < 65 years than in the subgroup aged ≥ 65 years (Supplementary Figure S2 and Supplementary Table S2). Regardless of smoking, alcohol consumption, and history of CVD and cancer, a higher serum GGT level was generally associated with a higher risk of mortality despite showing significant interaction (all *p* for interaction < 0.001). The higher mortality related to a higher GGT level was more pronounced in alcohol drinkers than non-drinkers. The association between GGT and mortality was more prominent in current smokers who smoked less than 20 pack-years.

### Stratified analysis: disease-specific mortality by covariates

A greater association between higher serum GGT level and increased risk of CVD-, cancer-, respiratory disease-, and liver disease-specific mortality was noted among men and younger participants (all *p* for interaction < 0.001) (Supplementary Figure S3-S6 and Supplementary Table S2). A higher serum GGT level was more prominently associated with a higher risk of CVD-specific mortality among in those without previous CVD history (*p* for interaction < 0.001) (Supplementary Figure S3). In terms of cancer-specific mortality, a higher serum GGT level was more strongly associated with a higher risk of cancer mortality in cancer patients than those without cancer history (*p* for interaction < 0.001) (Supplementary Figure S4).

### Sensitivity analysis

The association between GGT and all-cause and disease-specific mortality was consistent after adjustment for FLI and AST/ALT ratio (Supplementary Table S3). The analyses with 2 and 4 years of lag time also showed consistent findings even though such association was attenuated (Supplementary Table S4). The association of quintiles of GGT and mortality supported our main results (Supplementary Table S5). Among women, all-cause mortality in quintile 2 (HR = 0.95, 95% CI 0.93–0.97) and quintile 3 (HR = 0.95, 95% CI 0.93–0.96) was lower than that of quintile 1 which was consistent with findings with tertiles.

## Discussion

This is the first study, to our knowledge, to show the associations between GGT level and a broad range of causes of death, including less frequently evaluated outcomes, based on a nationwide, population-based cohort. Positive association between GGT and death was observed across all causes of mortality evaluated, with dose–response relationships for most types of death, even within the normal limits of GGT. A greater association between GGT and disease-specific mortality was noted among men and younger aged participants, suggesting the usefulness of GGT as a prognostic marker in these subgroups.

The association of GGT and heart disease has been reported by several studies. GGT is present in coronary atherosclerotic plaques and induces oxidation of low-density lipoprotein cholesterol within the diseased vessel wall, implicating its role in the progression of atherosclerosis^14–16^. Considerable evidence from epidemiolocal studies support the positive correlation of GGT with the degree of coronary atherosclerosis and incident CVD risk^8,17^. In addition, high GGT was associated with high-risk features of atherosclerosis detected by coronary computed tomography and with poorer cardiac outcomes defined as a composite of all-cause death, myocardial infarction, unstable angina requiring hospitalization, or coronary revascularization in subjects without significant alcohol consumption^18^. Similarly, GGT was positively associated with the risk of incident heart failure in prospective cohort studies^19,20^ and was an independent predictor of death or heart transplantation in patients with heart failure^21^. Our analyses also confirmed the relationship of GGT to heart disease mortality, even with adjustment for several known cardiometabolic risk factors and among those without previous CVD.

There is less evidence for the relationship of GGT to cancer-associated prognosis. Although studies have reported that GGT expression is increased in various cancers^2,22^ and its pro-oxidant activity might promote tumor progression by modulating cell proliferation and apoptosis^23^, it is uncertain whether GGT has causal roles in tumor biology or is merely an epiphenomenon associated with carcinogenesis. However, several studies have demonstrated that GGT positively correlated with the development of various cancers including breast, lung, endometrium, gastrointestinal tract, and liver^24–28^. Similarly, our analyses showed GGT to be positively associated with overall cancer mortality. Further studies are needed to validate these observed associations and the underlying mechanisms.

Results for respiratory disease-specific mortality types have been investigated more rarely. A cohort study based on male construction workers reported no significant association between GGT and death from respiratory causes after adjusting for alcohol^7^, whereas another study has reported that GGT may predict non-smoking subjects at high risk of decreased pulmonary function and/or chronic obstructive pulmonary disease^29^. Furthermore, high GGT level was associated with longer hospital stay and in-hospital mortality among patients with severe acute respiratory syndrome coronavirus 2^30,31^. Considering the relationship of GGT elevation with smoking^32,33^, a greater association between serum GGT level and respiratory disease-specific mortality in smokers than non-smokers suggests the possibility of residual confounding associated with smoking status. However, stratified analysis in non-smokers revealed consistent results in this study. Further studies to explain the association between respiratory disease-related mortality and GGT are warranted.

This study revealed the association of GGT to liver-related mortality by showing a greater than sixfold increased risk of death for the highest GGT group, even in subjects without underlying liver disease such as chronic viral hepatitis or cirrhosis. Because GGT has been strongly associated with both alcoholic and nonalcoholic fatty liver disease^2,34,35^, we adjusted for several known liver disease-associated factors of alcohol use, BMI, metabolic dysfunction, and ALT. The positive association of GGT and liver-related mortality was consistently shown in both non-drinkers and drinkers and was not affected by obesity and metabolic risk factors, suggesting that GGT can be a relevant indicator for liver-related mortality.

It is not clear why high GGT levels would be associated with increased disease-specific mortality. An association with oxidative stress is the hypothesis with the most support^1,2^. Active GGT was demonstrated in atherosclerotic plaques and may play a role in the development of reactive oxygen species^14,15^. In terms of cancer, GGT may provide a growth advantage to cancer cells via a GGT-dependent increased supply of cysteine, as well as protecting against apoptosis through upregulation of poly (adenosine diphosphate-ribose) polymerase activity^36–38^. Similarly, oxidative stress appears to be a plausible explanation for the association of GGT with respiratory disease. Lung inflammation induces the production of GGT by lung epithelial cells and secretion into epithelial lining fluid, which may paradoxically increase epithelial damage by decreasing glutathione concentrations^39–41^. In addition, although GGT lacks specificity, it is very sensitive for the detection of liver injury and strongly associated with alcoholic and nonalcoholic fatty liver disease. Further mechanistic studies are warranted to elucidate the role of GGT in the pathogenesis of the aforementioned diseases.

The dose–response association between GGT level and mortality was significant in men but less pronounced in women in this study. Women in our study had a significantly narrow range of GGT level along with the tertiles compared to men, resulting in trivial differences in risk factors across GGT tertile, possibly due to the short 8.3-year follow-up not allowing sufficient time to properly determine fatalities. However, our results are consistent with previous studies showing that women had weaker associations of GGT level with all-cause and CVD-specific mortality than men^42,43^. A stronger association between arterial stiffness and GGT level in men also has been reported in a health checkup cohort study^44^. In addition, a significant association between blood pressure and GGT level in men, but not women, has been reported in a general population-based cohort study^45^. These findings suggest that GGT might be considered a prognostic marker for mortality, especially in men, although the mechanism of sex-divergent outcomes should be evaluated in further studies.

Various studies have shown that the prognostic value of GGT was stronger in a younger age group than an elderly one. An Austrian cohort study showed hazard ratios for log GGT level and CVD mortality of 2.03 and 2.60, respectively, in younger (< 60 years) men and women, which decreased to 1.42 and 1.52, respectively, in elderly (≥ 60 years) men and women^9^. A nested case–control study also reported that serum GGT could predict CVD mortality in participants younger than 70 years but had limited usefulness for risk stratification in those older than 70 years^46^. On the contrary, some recent studies have shown positive associations between GGT and mortality even in elderly subjects with the mean age of 70 years^47,48^. Because GGT activities are age-dependent and the upper limit of normal defined as the 95th percentile for GGT increases up to the age of 70 in men and throughout life in women^49,50^, differences in risk factors according to GGT level might be attenuated in elderly subjects. However, even though the positive associations between GGT level and all-cause, CVD, cancer, respiratory and liver mortality were weaker in elderly (≥ 65 years) versus younger (< 65 years) subjects in this study, the predictive value of GGT remained significant in both groups, demonstrating its use in risk assessment.

There are several limitations in our study. First, there is the possibility of unmeasured confounding, such as presence of non-alcoholic fatty liver disease (NAFLD), since elevated GGT level is frequently accompanied with NAFLD. As ultrasonography is not employed in the NHIS screening program, NAFLD could not be precisely assessed by ultrasound examination. However, we tried to adjust for possible confounders thoroughly, including metabolic risk factors and CCI score. Second, the 8.3-year duration of follow-up might limit the ability to capture related mortality, especially from liver disease. This limitation is balanced by the benefits of our nationwide, population-based study design. Third, our study could not capture the impact of longitudinal changes of GGT level on outcomes because we used a single measurement of GGT level. Finally, given the nature of this epidemiologic study nature, the mechanisms driving the association between GGT and mortality could not be addressed directly. At present, it is unclear whether high GGT has a causal role or is just an epiphenomenon of risk factors clustered in population with high GGT levels. Further studies are warranted to validate and elucidate the underlying mechanisms for our findings.

In conclusion, GGT is an independent predictor of overall and disease-specific mortality, with a dose–response relationship in a general population. The underlying mechanism and clinical implications of GGT-based prognostication warrant further investigation.

## Supplementary Information

Supplementary Information.

## Data Availability

The data that support the findings of this study are available from the NHIS of Korea database. Restrictions apply to the availability of these data, which were used under license for this study. Data are available at https://nhiss.nhis.or.kr/bd/ay/bdaya001iv.do with the permission of the NHIS.
