# Supplementary material for: Gamma-glutamyl transferase and risk of all-cause and disease-specific mortality: a nationwide cohort study
Source: Sci Rep. 2023 Jan 31;13:1751. doi: 10.1038/s41598-022-25970-0 (PMC9888340; doi:10.1038/s41598-022-25970-0)
Supplement: Supplementary file 1 — Supplementary Information. [file 41598_2022_25970_MOESM1_ESM.docx]

**Supplementary Table S1.** **Variables with missing information**

| Variables | N of missing | % |
| --- | --- | --- |
| Sex | 0 | 0 |
| Income | 244,016 | 2.43 |
| BMI | 6,039 | 0.06 |
| Systolic BP | 2,609 | 0.03 |
| Diastolic BP | 2,640 | 0.03 |
| Fasting glucose | 2,107 | 0.02 |
| Total cholesterol | 1,872 | 0.02 |
| AST | 2,080 | 0.02 |
| ALT | 2,100 | 0.02 |
| GGT | 2,097 | 0.02 |
| Waist Circumference | 3,555 | 0.03 |
| Triglyceride | 2,632 | 0.03 |
| HDL -C | 3,908 | 0.04 |
| LDL -C | 4,122 | 0.04 |
| Serum Creatinine | 2,685 | 0.03 |
| Smoking | 65,231 | 0.64 |
| Drinking | 160,625 | 1.58 |
| Regular exercise | 209,705 | 2.08 |
| Estimated GFR | 3,200 | 0.03 |
| Fatty Liver Index | 11,715 | 0.11 |

BMI, body mass index; BP, blood pressure; HDL-C, high density lipoprotein-cholesterol; LDL-C, low density lipoprotein -cholesterol; TG, triglyceride; AST, aspartate transaminase; ALT, alanine transferase; GGT, gamma-glutamyl transferase; GFR, glomerular filtration rate

**Supplementary Table S2. Number of population and death by covariates**

|  |  | All-cause mortality | CVD-specific mortality | Cancer-specific mortality | Respiratory disease-specific mortality | Liver disease-specific mortality |
| --- | --- | --- | --- | --- | --- | --- |
|  | Total number (N) | Death events (N) | | | | |
| Sex |  |  |  |  |  |  |
| Male | 5,277,397 | 295,567 | 52,134 | 109,565 | 29,553 | 21,079 |
| Female | 4,409,669 | 165,132 | 38,423 | 52,927 | 14,091 | 5,747 |
| Age, years |  |  |  |  |  |  |
| <40 | 3,024,064 | 16,295 | 2,059 | 4,078 | 135 | 1,022 |
| 40-64 | 5,390,738 | 150,593 | 22,991 | 67,082 | 6,201 | 14,271 |
| ≥65 | 1,272,264 | 293,811 | 65,507 | 91,332 | 37,308 | 11,533 |
| Smoking status |  |  |  |  |  |  |
| No | 7,157,808 | 334,920 | 68,728 | 114,440 | 34,210 | 16,195 |
| Yes | 2,529,258 | 125,779 | 21,829 | 48,052 | 9,434 | 10,631 |
| Current Smoking |  |  |  |  |  |  |
| <20 pack years | 1,686,091 | 48,356 | 8,792 | 15,811 | 3,211 | 4,324 |
| ≥20 pack years | 843,167 | 77,423 | 13,037 | 32,241 | 6,223 | 6,307 |
| Drinking |  |  |  |  |  |  |
| None | 4,991,425 | 291,699 | 62,874 | 96,367 | 31,084 | 11,908 |
| Mild to moderate | 3,867,631 | 126,602 | 21,422 | 49,939 | 9,553 | 9,745 |
| Heavy | 828,010 | 42,398 | 6,261 | 16,186 | 3,007 | 5,173 |
| History of CVD |  |  |  |  |  |  |
| No | 9,509,127 | 419,768 | 78,026 | 153,178 | 38,842 | 25,752 |
| Yes | 177,939 | 40,931 | 12,531 | 9,314 | 4,802 | 1,074 |
| History of cancer |  |  |  |  |  |  |
| No | 9,538,474 | 434,262 | 88,280 | 78,930 | 22,466 | 25,513 |
| Yes | 148,592 | 26,437 | 2,277 | 83,562 | 21,178 | 1,313 |

CVD, cardiovascular disease

**Supplementary Table S3. All-cause and disease-specific mortality by tertiles of gamma-glutamyl transferase with additional adjustment for fatty liver index and AST/ALT ratio**

|  | N | Event | Duration | IR,  per 1000 PY | Model 4 | Model 5 |
| --- | --- | --- | --- | --- | --- | --- |
| Total |  |  |  |  | HR (95% CI) | HR (95% CI) |
| All-cause mortality | |  |  |  |  |  |
| Tertile 1 | 3,116,836 | 132,733 | 25663193.16 | 5.2 | 1 (Ref.) | 1 (Ref.) |
| Tertile 2 | 3,330,242 | 145,927 | 27415683.45 | 5.3 | 1.02 (1.02 - 1.03) | 1.03 (1.02 - 1.03) |
| Tertile 3 | 3,239,988 | 182,039 | 26528308.89 | 6.9 | 1.20 (1.19 - 1.21) | 1.20 (1.19 - 1.21) |
| CVD-specific mortality |  |  |  |  |  |  |
| Tertile 1 | 3,116,836 | 25,315 | 25663193.16 | 1.0 | 1 (Ref.) | 1 (Ref.) |
| Tertile 2 | 3,330,242 | 29,733 | 27415683.45 | 1.1 | 1.05 (1.03 - 1.07) | 1.04 (1.03 - 1.06) |
| Tertile 3 | 3,239,988 | 35,509 | 26528308.89 | 1.3 | 1.19 (1.16 - 1.21) | 1.16 (1.14 - 1.19) |
| Cancer-specific mortality | |  |  |  |  |  |
| Tertile 1 | 3,116,836 | 45,280 | 25663193.16 | 1.8 | 1 (Ref.) | 1 (Ref.) |
| Tertile 2 | 3,330,242 | 52,132 | 27415683.45 | 1.9 | 1.07 (1.06 - 1.09) | 1.07 (1.06 - 1.09) |
| Tertile 3 | 3,239,988 | 65,080 | 26528308.89 | 2.5 | 1.31 (1.29 - 1.33) | 1.31 (1.29 - 1.33) |
| Respiratory disease-specific mortality | |  |  |  |  |  |
| Tertile 1 | 3,116,836 | 15,323 | 25663193.16 | 0.6 | 1 (Ref.) | 1 (Ref.) |
| Tertile 2 | 3,330,242 | 14,146 | 27415683.45 | 0.5 | 1.08 (1.05 - 1.10) | 1.06 (1.03 - 1.09) |
| Tertile 3 | 3,239,988 | 14,175 | 26528308.89 | 0.5 | 1.25 (1.22 - 1.29) | 1.19 (1.15 - 1.22) |
| Liver disease-related mortality | |  |  |  |  |  |
| Tertile 1 | 3,116,836 | 2,851 | 25663193.16 | 0.1 | 1 (Ref.) | 1 (Ref.) |
| Tertile 2 | 3,330,242 | 4,967 | 27415683.45 | 0.2 | 1.74 (1.65 - 1.82) | 1.74 (1.66 - 1.82) |
| Tertile 3 | 3,239,988 | 19,008 | 26528308.89 | 0.7 | 5.82 (5.56 - 6.10) | 5.86 (5.59 - 6.14) |
| Male |  |  |  |  | HR (95% CI) | HR (95% CI) |
| All-cause mortality | |  |  |  |  |  |
| Tertile 1 | 1,761,605 | 96,785 | 14426469.99 | 6.7 | 1 (Ref.) | 1 (Ref.) |
| Tertile 2 | 1,736,845 | 90,199 | 14226226.51 | 6.3 | 1.05 (1.04 - 1.06) | 1.06 (1.04 - 1.07) |
| Tertile 3 | 1,778,947 | 108,583 | 14485267.72 | 7.5 | 1.31 (1.30 - 1.33) | 1.32 (1.30 - 1.33) |
| CVD-specific mortality | |  |  |  |  |  |
| Tertile 1 | 1,761,605 | 17,285 | 14426469.99 | 1.2 | 1 (Ref.) | 1 (Ref.) |
| Tertile 2 | 1,736,845 | 16,918 | 14226226.51 | 1.2 | 1.08 (1.05 - 1.10) | 1.07 (1.05 - 1.10) |
| Tertile 3 | 1,778,947 | 17,931 | 14485267.72 | 1.2 | 1.22 (1.18 - 1.25) | 1.20 (1.17 - 1.24) |
| Cancer-specific mortality | | |  |  |  |  |
| Tertile 1 | 1,761,605 | 33,878 | 14426469.99 | 2.3 | 1 (Ref.) | 1 (Ref.) |
| Tertile 2 | 1,736,845 | 34,022 | 14226226.51 | 2.4 | 1.11 (1.10 - 1.13) | 1.12 (1.10 - 1.13) |
| Tertile 3 | 1,778,947 | 41,665 | 14485267.72 | 2.9 | 1.48 (1.45 - 1.51) | 1.49 (1.46 - 1.51) |
| Respiratory disease-specific mortality | | | |  |  |  |
| Tertile 1 | 1,761,605 | 12,103 | 14426469.99 | 0.8 | 1 (Ref.) | 1 (Ref.) |
| Tertile 2 | 1,736,845 | 9,243 | 14226226.51 | 0.6 | 1.12 (1.09 - 1.15) | 1.10 (1.07 - 1.14) |
| Tertile 3 | 1,778,947 | 8,207 | 14485267.72 | 0.6 | 1.28 (1.24 - 1.33) | 1.22 (1.17 - 1.27) |
| Liver disease-related mortality | | |  |  |  |  |
| Tertile 1 | 1,761,605 | 2,290 | 14426469.99 | 0.2 | 1 (Ref.) | 1 (Ref.) |
| Tertile 2 | 1,736,845 | 3,766 | 14226226.51 | 0.3 | 1.88 (1.78 - 1.99) | 1.89 (1.79 - 2.00) |
| Tertile 3 | 1,778,947 | 15,023 | 14485267.72 | 1.0 | 7.05 (6.68 - 7.44) | 7.09 (6.72 - 7.48) |
| Female |  |  |  |  | HR (95% CI) | HR (95% CI) |
| All-cause mortality | |  |  |  |  |  |
| Tertile 1 | 1,355,231 | 35,948 | 11236723.17 | 3.2 | 1 (Ref.) | 1 (Ref.) |
| Tertile 2 | 1,593,397 | 55,728 | 13189456.94 | 4.2 | 0.97 (0.96 - 0.99) | 0.97 (0.96 - 0.98) |
| Tertile 3 | 1,461,041 | 73,456 | 12043041.18 | 6.1 | 1.06 (1.05 - 1.08) | 1.05 (1.03 - 1.06) |
| CVD-specific mortality | |  |  |  |  |  |
| Tertile 1 | 1,355,231 | 8,030 | 11236723.17 | 0.7 | 1 (Ref.) | 1 (Ref.) |
| Tertile 2 | 1,593,397 | 12,815 | 13189456.94 | 1.0 | 0.99 (0.96 - 1.01) | 0.98 (0.95 - 1.01) |
| Tertile 3 | 1,461,041 | 17,578 | 12043041.18 | 1.5 | 1.15 (1.12 - 1.19) | 1.11 (1.08 - 1.140) |
| Cancer-specific mortality | | |  |  |  |  |
| Tertile 1 | 1,355,231 | 11,402 | 11236723.17 | 1.0 | 1 (Ref.) | 1 (Ref.) |
| Tertile 2 | 1,593,397 | 18,110 | 13189456.94 | 1.4 | 1.02 (0.99 - 1.04) | 1.02 (0.99 - 1.05) |
| Tertile 3 | 1,461,041 | 23,415 | 12043041.18 | 1.9 | 1.11 (1.08 - 1.14) | 1.12 (1.09 - 1.14) |
| Respiratory disease-specific mortality | | | |  |  |  |
| Tertile 1 | 1,355,231 | 3,220 | 11236723.17 | 0.3 | 1 (Ref.) | 1 (Ref.) |
| Tertile 2 | 1,593,397 | 4,903 | 13189456.94 | 0.4 | 1.02 (0.97 - 1.06) | 1.00 (0.96 - 1.05) |
| Tertile 3 | 1,461,041 | 5,968 | 12043041.18 | 0.5 | 1.15 (1.10 - 1.21) | 1.09 (1.04 - 1.14) |
| Liver disease-related mortality | | |  |  |  |  |
| Tertile 1 | 1,355,231 | 561 | 11236723.17 | 0.0 | 1 (Ref.) | 1 (Ref.) |
| Tertile 2 | 1,593,397 | 1,201 | 13189456.94 | 0.1 | 1.38 (1.25 - 1.53) | 1.38 (1.25 - 1.53) |
| Tertile 3 | 1,461,041 | 3,985 | 12043041.18 | 0.3 | 3.46 (3.14 - 3.80) | 3.50 (3.19 - 3.85) |

IR, incidence rate; PY, person year; CVD, cardiovascular diseases, HR, hazard ratio; CI, confidence interval; AST, aspartate aminotransferase; ALT, alanine transaminase

Model 4 was adjusted for age, sex, smoking status, alcohol consumption, physical activity, low income, body mass index, alanine aminotransferase, diabetes mellitus, hypertension, dyslipidemia, Charlson comorbidity index score, and fatty liver index.

Model 5 was adjusted for age, sex, smoking status, alcohol consumption, physical activity, low income, body mass index, diabetes mellitus, hypertension, dyslipidemia, Charlson comorbidity index score, fatty liver index, and AST/ALT ratio.

**Supplementary Table S4. Sensitivity analyses with 2 and 4 years of lag time**

|  | N | Event | IR, per 1000 PY | Model 1 | Model 2 | Model 3 |
| --- | --- | --- | --- | --- | --- | --- |
| **2-year lag** |  |  |  | HR (95% CI) | HR (95% CI) | HR (95% CI) |
| **All-cause mortality** | |  |  |  |  |  |
| Tertile 1 | 3,107,132 | 123,029 | 5.5 | 1 (Ref.) | 1 (Ref.) | 1 (Ref.) |
| Tertile 2 | 3,320,225 | 135,910 | 5.6 | 1.04 (1.04 - 1.05) | 1.02 (1.02 - 1.03) | 1.05 (1.04 - 1.06) |
| Tertile 3 | 3,226,826 | 168,877 | 7.2 | 1.37 (1.36 - 1.38) | 1.29 (1.28 - 1.30) | 1.31 (1.30 - 1.32) |
| **CVD-specific mortality** | |  |  |  |  |  |
| Tertile 1 | 3,107,132 | 23,528 | 1.0 | 1 (Ref.) | 1 (Ref.) | 1 (Ref.) |
| Tertile 2 | 3,320,225 | 27,717 | 1.2 | 1.11 (1.09 - 1.13) | 1.10 (1.08 - 1.12) | 1.07 (1.05 - 1.09) |
| Tertile 3 | 3,226,826 | 32,978 | 1.4 | 1.40 (1.38 - 1.43) | 1.36 (1.34 - 1.39) | 1.28 (1.26 - 1.31) |
| **Cancer-specific mortality** | |  |  |  |  |  |
| Tertile 1 | 3,107,132 | 41,634 | 1.8 | 1 (Ref.) | 1 (Ref.) | 1 (Ref.) |
| Tertile 2 | 3,320,225 | 48,413 | 2.0 | 1.09 (1.08 - 1.11) | 1.06 (1.05 - 1.08) | 1.08 (1.06 - 1.09) |
| Tertile 3 | 3,226,826 | 60,200 | 2.6 | 1.42 (1.40 - 1.44) | 1.32 (1.30 - 1.34) | 1.34 (1.32 - 1.36) |
| **Respiratory disease-specific mortality** | | |  |  |  |  |
| Tertile 1 | 3,107,132 | 14,607 | 0.6 | 1 (Ref.) | 1 (Ref.) | 1 (Ref.) |
| Tertile 2 | 3,320,225 | 13,515 | 0.6 | 0.99 (0.97 - 1.02) | 0.99 (0.97 - 1.01) | 1.10 (1.07 - 1.13) |
| Tertile 3 | 3,226,826 | 13,563 | 0.6 | 1.18 (1.15 - 1.21) | 1.15 (1.12 - 1.18) | 1.38 (1.34 - 1.41) |
| **Liver disease-related mortality** | | |  |  |  |  |
| Tertile 1 | 3,107,132 | 2,665 | 0.1 | 1 (Ref.) | 1 (Ref.) | 1 (Ref.) |
| Tertile 2 | 3,320,225 | 4,638 | 0.2 | 1.70 (1.62 - 1.78) | 1.67 (1.59 - 1.76) | 1.76 (1.67 - 1.85) |
| Tertile 3 | 3,226,826 | 17,494 | 0.8 | 6.73 (6.46 - 7.03) | 6.00 (5.75 - 6.26) | 6.42 (6.15 - 6.71) |
| **4-year lag** |  |  |  |  |  |  |
| **All-cause mortality** | |  |  |  |  |  |
| Tertile 1 | 3,083,979 | 99,876 | 6.1 | 1 (Ref.) | 1 (Ref.) | 1 (Ref.) |
| Tertile 2 | 3,295,621 | 111,306 | 6.4 | 1.05 (1.04 - 1.05) | 1.03 (1.02 - 1.04) | 1.04 (1.04 - 1.05) |
| Tertile 3 | 3,195,591 | 137,642 | 8.2 | 1.37 (1.35 - 1.38) | 1.28 (1.27 - 1.29) | 1.29 (1.28 - 1.31) |
| **CVD-specific mortality** | |  |  |  |  |  |
| Tertile 1 | 3,083,979 | 19,270 | 1.2 | 1 (Ref.) | 1 (Ref.) | 1 (Ref.) |
| Tertile 2 | 3,295,621 | 22,927 | 1.3 | 1.11 (1.09 - 1.13) | 1.10 (1.08 - 1.12) | 1.07 (1.05 - 1.09) |
| Tertile 3 | 3,195,591 | 27,096 | 1.6 | 1.40 (1.37 - 1.42) | 1.35 (1.33 - 1.38) | 1.27 (1.24 - 1.29) |
| **Cancer-specific mortality** | |  |  |  |  |  |
| Tertile 1 | 3,083,979 | 33,021 | 2.0 | 1 (Ref.) | 1 (Ref.) | 1 (Ref.) |
| Tertile 2 | 3,295,621 | 38,957 | 2.2 | 1.10 (1.08 - 1.12) | 1.07 (1.05 -1.08) | 1.08 (1.06 - 1.10) |
| Tertile 3 | 3,195,591 | 48,346 | 2.9 | 1.42 (1.40 - 1.44) | 1.31 (1.29 - 1.33) | 1.32 (1.30 - 1.35) |
| **Respiratory disease-specific mortality** | | |  |  |  |  |
| Tertile 1 | 3,083,979 | 12,563 | 0.8 | 1 (Ref.) | 1 (Ref.) | 1 (Ref.) |
| Tertile 2 | 3,295,621 | 11,801 | 0.7 | 1.00 (0.98 - 1.03) | 0.99 (0.97 - 1.02) | 1.09 (1.07 - 1.12) |
| Tertile 3 | 3,195,591 | 11,848 | 0.7 | 1.18 (1.15 - 1.21) | 1.15 (1.12 - 1.18) | 1.36 (1.32 - 1.40) |
| **Liver disease-related mortality** | | |  |  |  |  |
| Tertile 1 | 3,083,979 | 2,145 | 0.1 | 1 (Ref.) | 1 (Ref.) | 1 (Ref.) |
| Tertile 2 | 3,295,621 | 3,777 | 0.2 | 1.70 (1.62 - 1.80) | 1.68 (1.59 - 1.77) | 1.75 (1.65 - 1.84) |
| Tertile 3 | 3,195,591 | 13,905 | 0.8 | 6.59 (6.29 - 6.90) | 5.84 (5.57 - 6.13) | 6.17 (5.88 - 6.48) |

PY, person year; CVD, cardiovascular diseases, HR, hazard ratio; CI, confidence interval

Model 1 was adjusted for age and sex; Model 2 was adjusted for smoking status, alcohol consumption, physical activity, and low income in addition to covariates in model 1; Model 3 was adjusted for body mass index, alanine aminotransferase, diabetes mellitus, hypertension, dyslipidemia, and Charlson comorbidity index score in addition to covariates in model 2.

**Supplementary Table S5. All-cause and disease-specific mortality by quintiles of gamma-glutamyl transferase**

| Quintiles^a^ | N | Event | IR  (per 1000 PY) | Model 1 | Model 2 | Model 3 |
| --- | --- | --- | --- | --- | --- | --- |
| **Total** |  |  |  |  |  |  |
| **All-cause mortality** | |  |  |  |  |  |
| Quintile 1 | 1,795,767 | 78,052 | 5.3 | 1 (Ref.) | 1 (Ref.) | 1 (Ref.) |
| Quintile 2 | 2,001,782 | 84,133 | 5.1 | 0.98 (0.98 - 0.99) | 0.98 (0.97 - 0.99) | 0.99 (0.99 - 1.01) |
| Quintile 3 | 1,995,390 | 86,764 | 5.3 | 1.03 (1.02 - 1.04) | 1.01 (0.99 - 1.02) | 1.05 (1.04 - 1.06) |
| Quintile 4 | 2,002,679 | 93,971 | 5.7 | 1.11 (1.10 - 1.12) | 1.08 (1.07 - 1.09) | 1.13 (1.11 - 1.14) |
| Quintile 5 | 1,891,448 | 117,779 | 7.6 | 1.53 (1.52 - 1.55) | 1.42 (1.41 - 1.44) | 1.46 (1.45 - 1.48) |
| **CVD-specific mortality** | |  |  |  |  |  |
| Quintile 1 | 1,795,767 | 14,586 | 1.0 | 1 (Ref.) | 1 (Ref.) | 1 (Ref.) |
| Quintile 2 | 2,001,782 | 16,597 | 1.0 | 1.05 (1.03 - 1.07) | 1.05 (1.02 - 1.07) | 1.03 (1.01 - 1.06) |
| Quintile 3 | 1,995,390 | 17,665 | 1.1 | 1.12 (1.10 - 1.15) | 1.11 (1.09 - 1.14) | 1.09 (1.06 - 1.11) |
| Quintile 4 | 2,002,679 | 19,427 | 1.2 | 1.22 (1.20 - 1.25) | 1.21 (1.18 - 1.24) | 1.16 (1.14 - 1.19) |
| Quintile 5 | 1,891,448 | 22,282 | 1.4 | 1.57 (1.54 - 1.61) | 1.52 (1.49 - 1.55) | 1.43 (1.40 - 1.46) |
| **Cancer-specific mortality** | |  |  |  |  |  |
| Quintile 1 | 1,795,767 | 26,036 | 1.8 | 1 (Ref.) | 1 (Ref.) | 1 (Ref.) |
| Quintile 2 | 2,001,782 | 29,678 | 1.8 | 1.03 (1.01 - 1.05) | 1.02 (0.99 - 1.03) | 1.03 (1.01 - 1.05) |
| Quintile 3 | 1,995,390 | 31,116 | 1.9 | 1.10 (1.08 - 1.12) | 1.06 (1.05 - 1.08) | 1.09 (1.08 - 1.11) |
| Quintile 4 | 2,002,679 | 33,869 | 2.1 | 1.19 (1.17 - 1.21) | 1.13 (1.11 - 1.15) | 1.18 (1.16 - 1.20) |
| Quintile 5 | 1,891,448 | 41,793 | 2.7 | 1.59 (1.57 - 1.62) | 1.47 (1.45 - 1.50) | 1.52 (1.49 - 1.54) |
| **Respiratory disease-specific mortality** | | |  |  |  |  |
| Quintile 1 | 1,795,767 | 9,506 | 0.6 | 1 (Ref.) | 1 (Ref.) | 1 (Ref.) |
| Quintile 2 | 2,001,782 | 8,903 | 0.5 | 0.94 (0.92 - 0.97) | 0.94 (0.91 - 0.97) | 1.02 (0.99 - 1.05) |
| Quintile 3 | 1,995,390 | 8,340 | 0.5 | 0.96 (0.94 - 0.99) | 0.96 (0.93 - 0.99) | 1.11 (1.07 - 1.14) |
| Quintile 4 | 2,002,679 | 8,337 | 0.5 | 1.02 (0.99 - 1.05) | 1.01 (0.98 - 1.05) | 1.23 (1.19 - 1.27) |
| Quintile 5 | 1,891,448 | 8,558 | 0.6 | 1.24 (1.20 - 1.28) | 1.20 (1.17 - 1.24) | 1.53 (1.48 - 1.58) |
| **Liver disease-related mortality** | | |  |  |  |  |
| Quintile 1 | 1,795,767 | 1,561 | 0.1 | 1 (Ref.) | 1 (Ref.) | 1 (Ref.) |
| Quintile 2 | 2,001,782 | 2,072 | 0.1 | 1.21 (1.13 - 1.29) | 1.19 (1.11 - 1.27) | 1.24 (1.16 - 1.33) |
| Quintile 3 | 1,995,390 | 2,943 | 0.2 | 1.81 (1.70 - 1.93) | 1.78 (1.67 - 1.90) | 1.93 (1.81 - 2.05) |
| Quintile 4 | 2,002,679 | 4,548 | 0.3 | 2.84 (2.68 - 3.01) | 2.79 (2.63 - 2.96) | 3.09 (2.91 - 3.28) |
| Quintile 5 | 1,891,448 | 15,702 | 1.0 | 10.54 (9.99 - 11.10) | 9.53 (9.02 - 10.06) | 10.55 (9.98 - 11.15) |
| **Male** |  |  |  |  |  |  |
| **All-cause mortality** | |  |  |  |  |  |
| Quintile 1 | 1,027,361 | 58,439 | 6.9 | 1 (Ref.) | 1 (Ref.) | 1 (Ref.) |
| Quintile 2 | 1,122,651 | 58,830 | 6.4 | 0.98 (0.97 - 0.99) | 0.98 (0.97 - 0.99) | 1.01 (0.99 - 1.02) |
| Quintile 3 | 1,033,012 | 53,541 | 6.3 | 1.03 (1.02 - 1.05) | 1.02 (1.01 - 1.03) | 1.08 (1.06 - 1.09) |
| Quintile 4 | 1,052,035 | 53,126 | 6.2 | 1.13 (1.11 - 1.14) | 1.10 (1.09 - 1.12) | 1.18 (1.16 - 1.19) |
| Quintile 5 | 1,042,338 | 71,631 | 8.5 | 1.69 (1.67 - 1.71) | 1.59 (1.57 - 1.61) | 1.65 (1.63 - 1.67) |
| **CVD-specific mortality** | |  |  |  |  |  |
| Quintile 1 | 1,027,361 | 10,346 | 1.2 | 1 (Ref.) | 1 (Ref.) | 1 (Ref.) |
| Quintile 2 | 1,122,651 | 10,761 | 1.2 | 1.04 (1.01 - 1.06) | 1.04 (1.01 - 1.06) | 1.02 (0.99 - 1.04) |
| Quintile 3 | 1,033,012 | 10,016 | 1.2 | 1.13 (1.10 - 1.16) | 1.13 (1.10 - 1.16) | 1.09 (1.06 - 1.12) |
| Quintile 4 | 1,052,035 | 9,742 | 1.1 | 1.23 (1.19 - 1.26) | 1.24 (1.20 - 1.27) | 1.17 (1.14 - 1.21) |
| Quintile 5 | 1,042,338 | 11,269 | 1.3 | 1.61 (1.56 - 1.65) | 1.57 (1.52 - 1.62) | 1.44 (1.40 -1.49) |
| **Cancer-specific mortality** | |  |  |  |  |  |
| Quintile 1 | 1,027,361 | 19,917 | 2.4 | 1 (Ref.) | 1 (Ref.) | 1 (Ref.) |
| Quintile 2 | 1,122,651 | 21,513 | 2.3 | 1.05 (1.03 - 1.07) | 1.04 (1.02 - 1.06) | 1.06 (1.04 - 1.08) |
| Quintile 3 | 1,033,012 | 20,263 | 2.4 | 1.14 (1.12 - 1.17) | 1.10 (1.08 - 1.13) | 1.15 (1.13 - 1.17) |
| Quintile 4 | 1,052,035 | 20,713 | 2.4 | 1.28 (1.26 - 1.31) | 1.22 (1.19 - 1.24) | 1.28 (1.25 - 1.30) |
| Quintile 5 | 1,042,338 | 27,159 | 3.2 | 1.86 (1.83 - 1.90) | 1.73 (1.69 - 1.76) | 1.80 (1.76 - 1.84) |
| **Respiratory disease-specific mortality** | | |  |  |  |  |
| Quintile 1 | 1,027,361 | 7,691 | 0.9 | 1 (Ref.) | 1 (Ref.) | 1 (Ref.) |
| Quintile 2 | 1,122,651 | 6,672 | 0.7 | 0.94 (0.91 - 0.97) | 0.94 (0.90 - 0.97) | 1.04 (1.01 - 1.08) |
| Quintile 3 | 1,033,012 | 5,465 | 0.6 | 0.97 (0.94 - 1.01) | 0.97 (0.94 - 1.01) | 1.18 (1.13 - 1.22) |
| Quintile 4 | 1,052,035 | 4,787 | 0.6 | 1.02 (0.98 - 1.06) | 1.02 (0.98 - 1.06) | 1.30 (1.25 - 1.35) |
| Quintile 5 | 1,042,338 | 4,938 | 0.6 | 1.28 (1.23 - 1.33) | 1.25 (1.20 - 1.30) | 1.60 (1.53 - 1.67) |
| **Liver disease-related mortality** | | |  |  |  |  |
| Quintile 1 | 1,027,361 | 1,270 | 0.2 | 1 (Ref.) | 1 (Ref.) | 1 (Ref.) |
| Quintile 2 | 1,122,651 | 1,676 | 0.2 | 1.24 (1.15 - 1.33) | 1.22 (1.13 - 1.31) | 1.29 (1.20 - 1.39) |
| Quintile 3 | 1,033,012 | 2,225 | 0.3 | 1.84 (1.72 - 1.97) | 1.82 (1.70 - 1.96) | 2.03 (1.89 - 2.18) |
| Quintile 4 | 1,052,035 | 3,488 | 0.4 | 3.06 (2.86 - 3.26) | 3.05 (2.85 - 3.26) | 3.49 (3.26 - 3.73) |
| Quintile 5 | 1,042,338 | 12,420 | 1.5 | 11.70 (11.04 - 12.40) | 10.70 (10.07 - 11.37) | 12.13 (11.41 - 12.91) |
| **Female** |  |  |  |  |  |  |
| **All-cause mortality** | |  |  |  |  |  |
| Quintile 1 | 768,406 | 19,613 | 3.1 | 1 (Ref.) | 1 (Ref.) | 1 (Ref.) |
| Quintile 2 | 879,131 | 25,303 | 3.5 | 0.94 (0.92 - 0.96) | 0.94 (0.92 - 0.96) | 0.95 (0.93 - 0.97) |
| Quintile 3 | 962,378 | 33,223 | 4.2 | 0.94 (0.92 - 0.95) | 0.93 (0.92 - 0.95) | 0.95 (0.93 - 0.96) |
| Quintile 4 | 950,644 | 40,845 | 5.2 | 0.98 (0.96 - 0.99) | 0.97 (0.96 - 0.99) | 0.99 (0.97 - 1.01) |
| Quintile 5 | 849,110 | 46,148 | 6.6 | 1.20 (1.18 - 1.22) | 1.18 (1.16 - 1.20) | 1.19 (1.17 - 1.21) |
| **CVD-specific mortality** | |  |  |  |  |  |
| Quintile 1 | 768,406 | 4,240 | 0.7 | 1 (Ref.) | 1 (Ref.) | 1 (Ref.) |
| Quintile 2 | 879,131 | 5,836 | 0.8 | 1.01 (0.98 - 1.06) | 1.01 (0.98 - 1.06) | 1.01 (0.97 - 1.05) |
| Quintile 3 | 962,378 | 7,649 | 1.0 | 1.00 (0.97 - 1.04) | 1.00 (0.96 - 1.04) | 0.99 (0.96 - 1.03) |
| Quintile 4 | 950,644 | 9,685 | 1.2 | 1.09 (1.05 - 1.13) | 1.08 (1.04 - 1.12) | 1.06 (1.02 - 1.10) |
| Quintile 5 | 849,110 | 11,013 | 1.6 | 1.38 (1.33 - 1.43) | 1.36 (1.31 - 1.41) | 1.35 (1.29 - 1.40) |
| **Cancer-specific mortality** | |  |  |  |  |  |
| Quintile 1 | 768,406 | 6,119 | 1.0 | 1 (Ref.) | 1 (Ref.) | 1 (Ref.) |
| Quintile 2 | 879,131 | 8,165 | 1.1 | 0.99 (0.96 - 1.02) | 0.99 (0.96 - 1.02) | 0.99 (0.96 - 1.02) |
| Quintile 3 | 962,378 | 10,853 | 1.4 | 1.02 (0.99 - 1.05) | 1.02 (0.98 - 1.05) | 1.01 (0.98 - 1.05) |
| Quintile 4 | 950,644 | 13,156 | 1.7 | 1.06 (1.03 - 1.10) | 1.06 (1.03 - 1.09) | 1.04 (1.01 - 1.08) |
| Quintile 5 | 849,110 | 14,634 | 2.1 | 1.26 (1.22 - 1.29) | 1.24 (1.20 - 1.28) | 1.20 (1.16 - 1.23) |
| **Respiratory disease-specific mortality** | | |  |  |  |  |
| Quintile 1 | 768,406 | 1,815 | 0.3 | 1 (Ref.) | 1 (Ref.) | 1 (Ref.) |
| Quintile 2 | 879,131 | 2,231 | 0.3 | 0.92 (0.87 - 0.98) | 0.92 (0.87 - 0.98) | 0.95 (0.89 - 1.01) |
| Quintile 3 | 962,378 | 2,875 | 0.4 | 0.90 (0.85 - 0.95) | 0.89 (0.84 - 0.95) | 0.95 (0.90 - 1.01) |
| Quintile 4 | 950,644 | 3,550 | 0.5 | 0.96 (0.91 - 1.02) | 0.96 (0.90 - 1.01) | 1.06 (0.99 - 1.12) |
| Quintile 5 | 849,110 | 3,620 | 0.5 | 1.13 (1.06 - 1.19) | 1.11 (1.05 - 1.17) | 1.30 (1.23 - 1.38) |
| **Liver disease-related mortality** | | |  |  |  |  |
| Quintile 1 | 768,406 | 291 | 0.0 | 1 (Ref.) | 1 (Ref.) | 1 (Ref.) |
| Quintile 2 | 879,131 | 396 | 0.1 | 1.00 (0.86 - 1.17) | 0.99 (0.85 - 1.16) | 1.01 (0.87 - 1.18) |
| Quintile 3 | 962,378 | 718 | 0.1 | 1.40 (1.22 - 1.60) | 1.38 (1.20 - 1.58) | 1.42 (1.24 - 1.63) |
| Quintile 4 | 950,644 | 1,060 | 0.1 | 1.77 (1.55 - 2.01) | 1.75 (1.53 - 1.99) | 1.82 (1.59 - 2.07) |
| Quintile 5 | 849,110 | 3,282 | 0.5 | 5.82 (5.17 - 6.57) | 5.59 (4.95 - 6.30) | 5.78 (5.12 - 6.53) |

PY, person year; CVD, cardiovascular diseases, HR, hazard ratio; CI, confidence interval

^a^Quintile 1 (< 20, and < 12), quintile 2 (21-27, and 12-14), quintile 3 (28-38, and 15-18), quintile 4 (39-62, and 19-26), and quintile 5 (≥ 63, and ≥ 27) were used for men and women, respectively.

Model 1 was adjusted for age and sex; Model 2 was adjusted for smoking status, alcohol consumption, physical activity, and low income in addition to covariates in model 1; Model 3 was adjusted for body mass index, alanine aminotransferase, diabetes mellitus, hypertension, dyslipidemia, and Charlson comorbidity index score in addition to covariates in model 2.

**Supplementary Figure S1. Restricted cubic spline curve for association between gamma-glutamyl transferase and mortality**

**
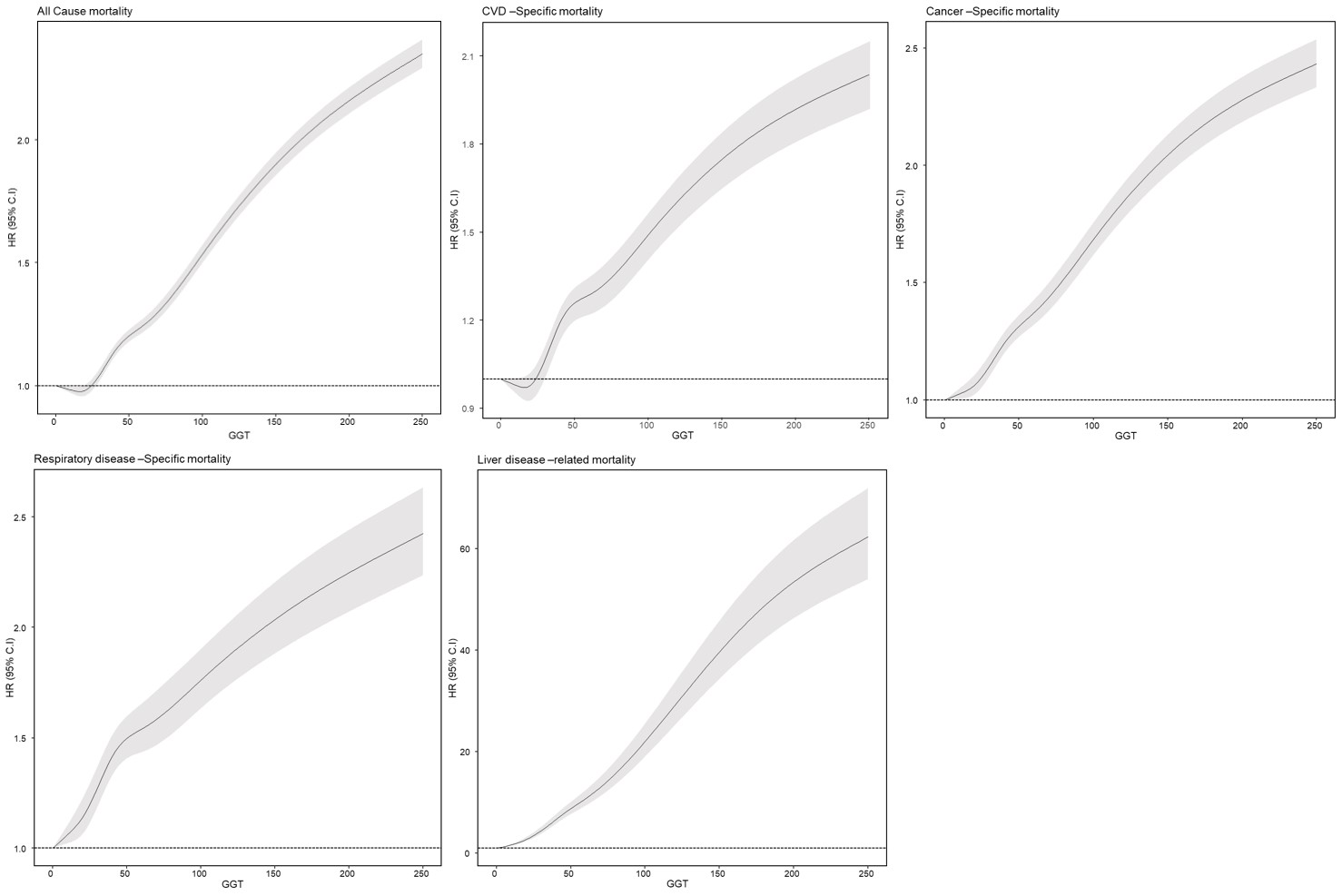
**

CVD, cardiovascular disease; GGT, gamma-glutamyl transferase

**Supplementary Figure S2. Stratification analysis by covariates for association between gamma-glutamyl transferase and all-cause mortality**

**
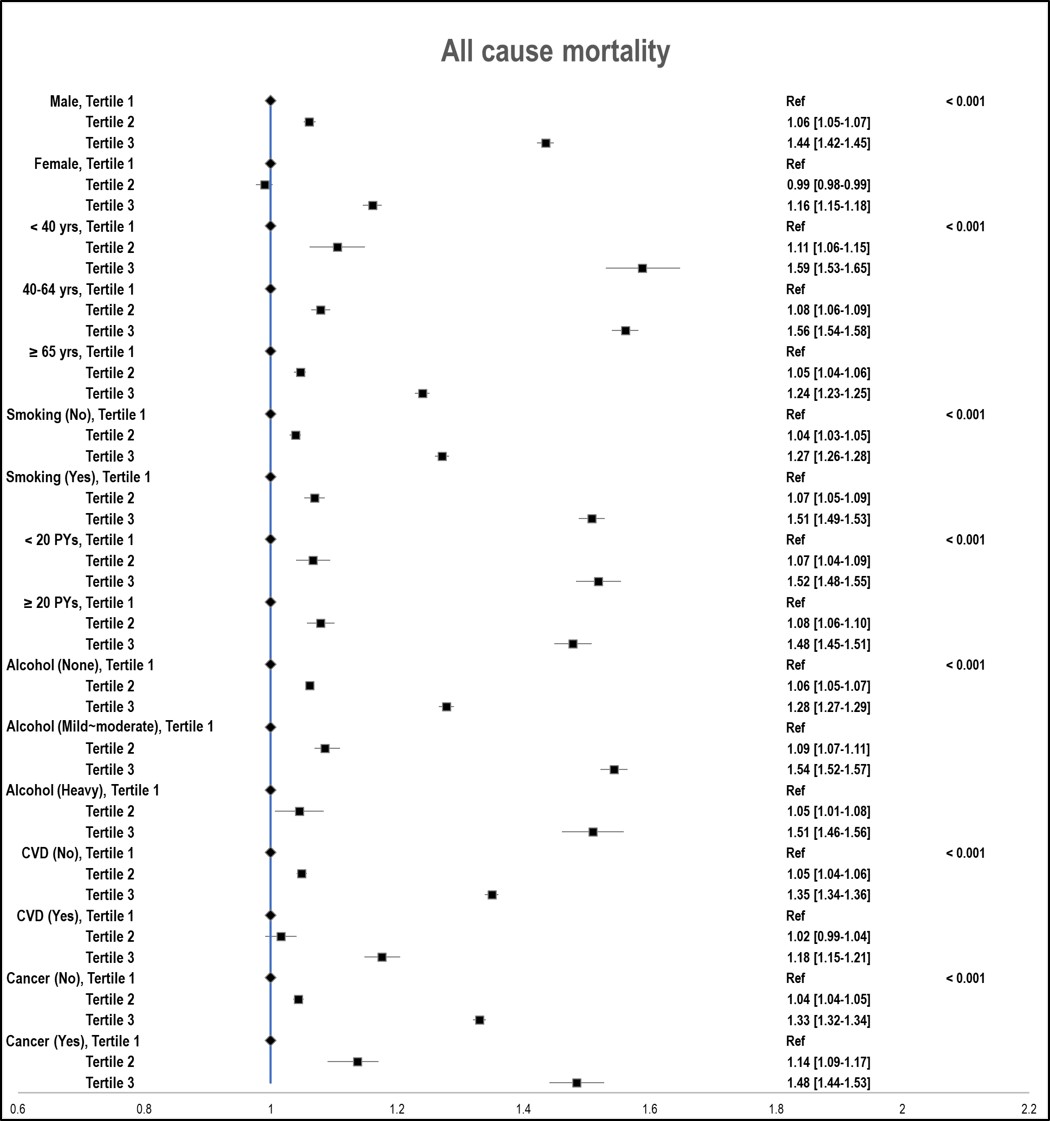
**

PYs, pack-years, CVD, cardiovascular disease

**Supplementary Figure S3. Stratification analysis by covariates for association between gamma-glutamyl transferase and cardiovascular disease-specific mortality**

**
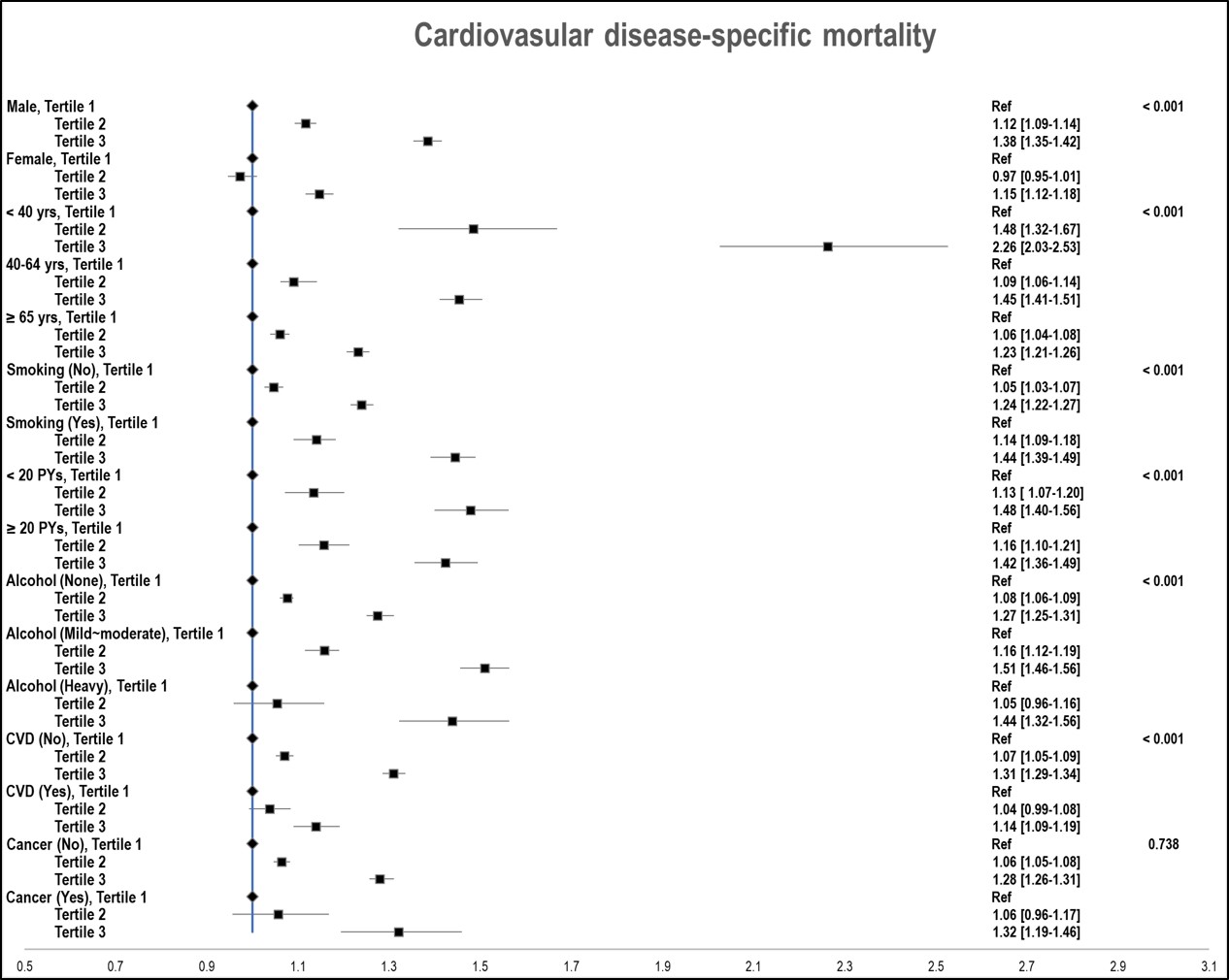
**

PYs, pack-years, CVD, cardiovascular disease

**Supplementary Figure S4. Stratification analysis by covariates for association between gamma-glutamyl transferase and cancer-specific mortality**

**
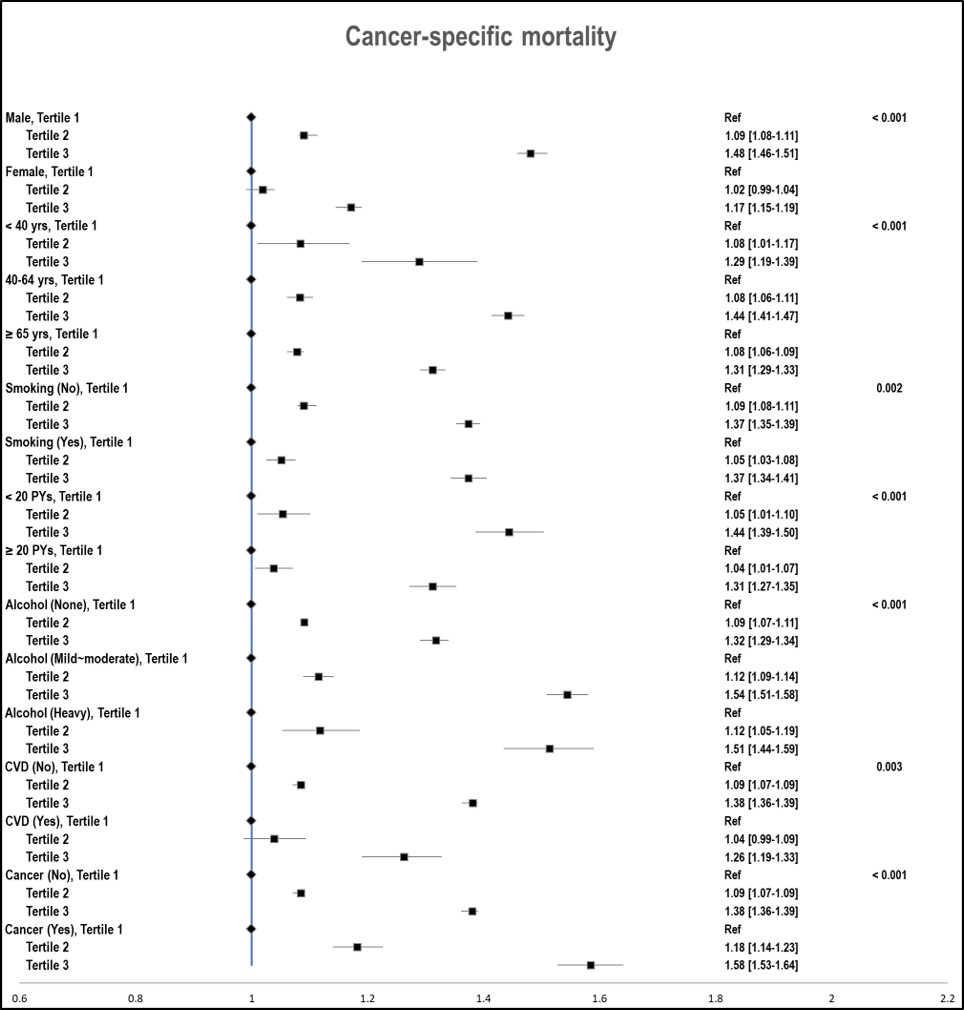
**

PYs, pack-years, CVD, cardiovascular disease

**Supplementary Figure S5. Stratification analysis by covariates for association between gamma-glutamyl transferase and respiratory disease-specific mortality**

**
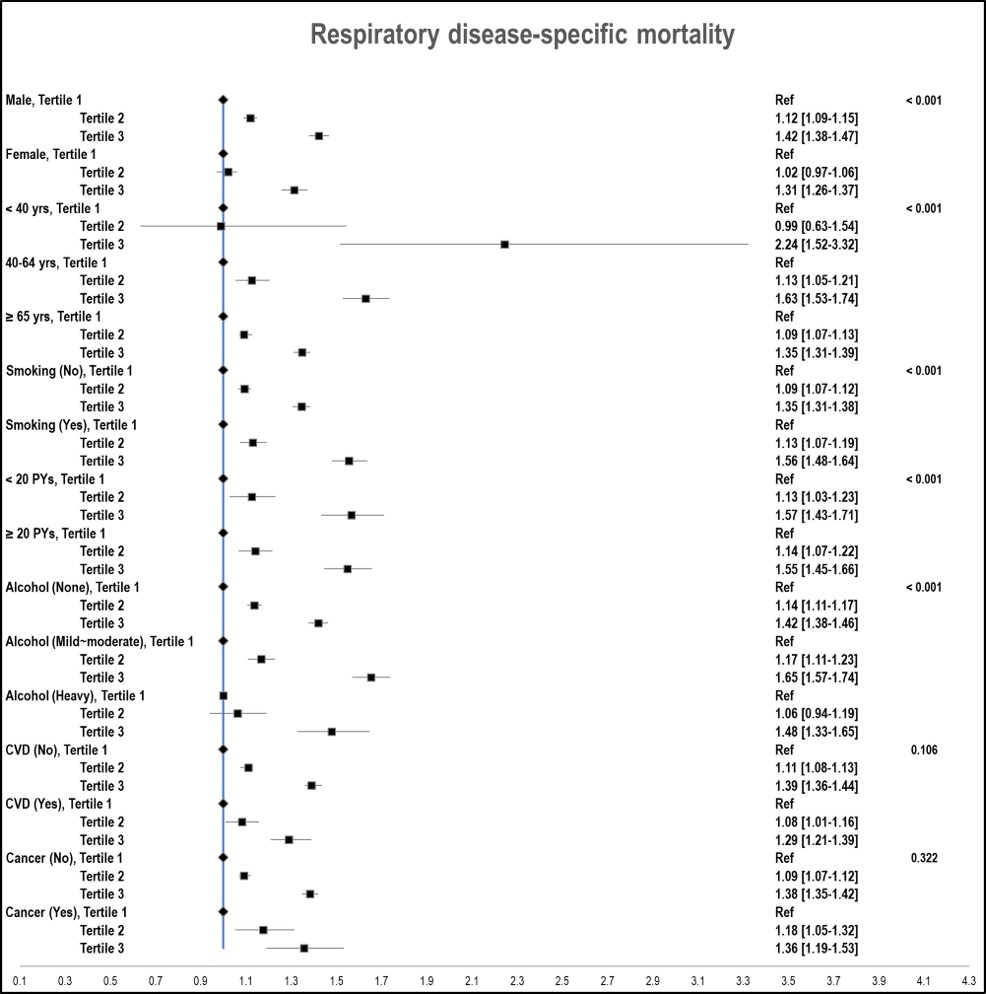
**

PYs, pack-years, CVD, cardiovascular disease

**Supplementary Figure S6. Stratification analysis by covariates for association between gamma-glutamyl transferase and liver disease-specific mortality**

**
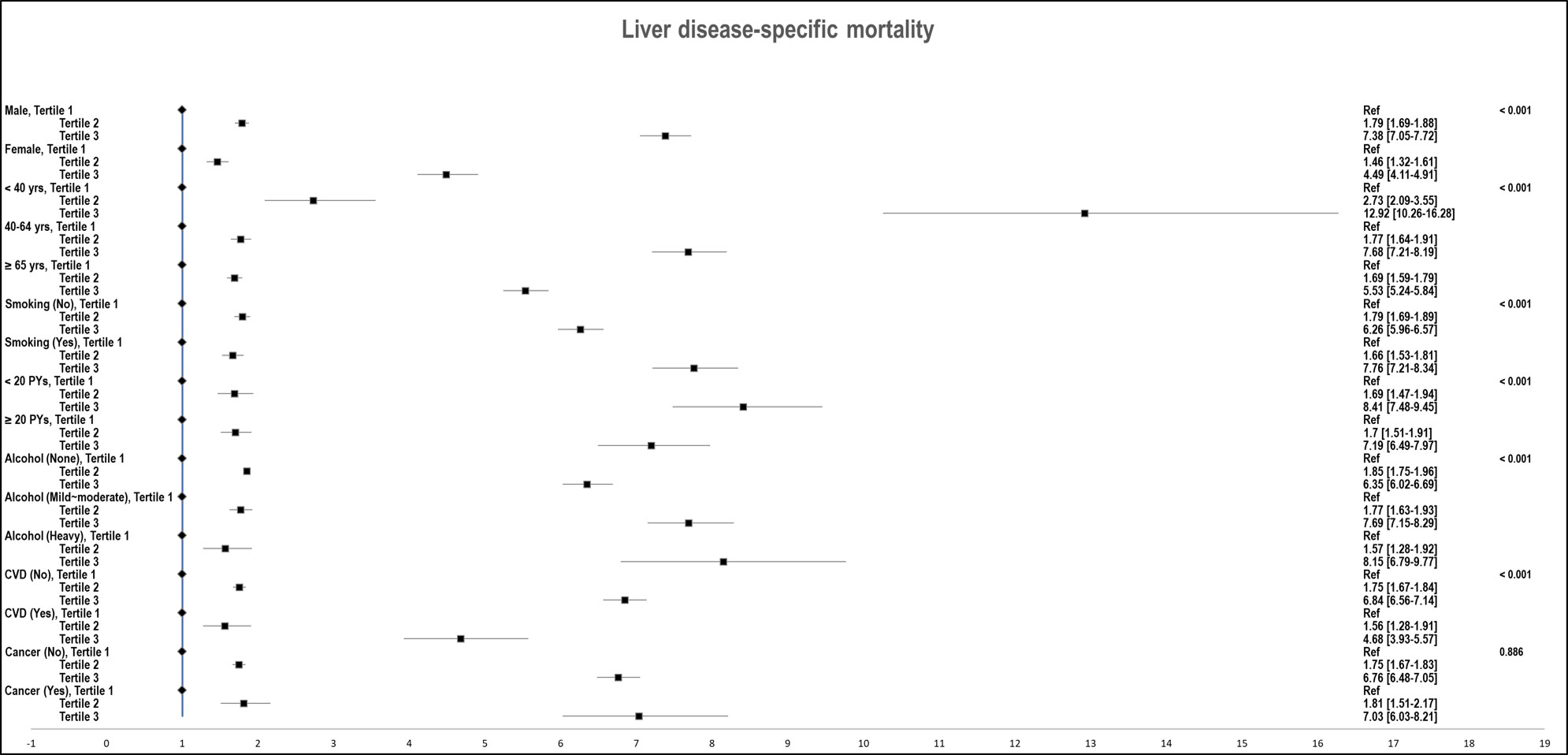
**

PYs, pack-years, CVD, cardiovascular disease
